# Simultaneous Action of Silymarin and Dopamine Enhances Defense Mechanisms Related to Antioxidants, Polyamine Metabolic Enzymes, and Tolerance to Cadmium Stress in *Phaseolus vulgaris*

**DOI:** 10.3390/plants11223069

**Published:** 2022-11-12

**Authors:** Awatif M. Abdulmajeed, Basmah M. Alharbi, Hesham F. Alharby, Amani M. Abualresh, Ghada A. Badawy, Wael M. Semida, Mostafa M. Rady

**Affiliations:** 1 Biology Department, Faculty of Science, University of Tabuk, Umluj 46429, Saudi Arabia; 2 Biology Department, Faculty of Science, University of Tabuk, Tabuk 71491, Saudi Arabia; 3 Department of Biological Sciences, Faculty of Science, King Abdulaziz University, Jeddah 21589, Saudi Arabia; 4 Horticulture Department, Faculty of Agriculture, Fayoum University, Fayoum 63514, Egypt; 5 Botany Department, Faculty of Agriculture, Fayoum University, Fayoum 63514, Egypt

**Keywords:** silymarin, dopamine, common bean, cadmium stress, anti-oxidative defense systems, abiotic stress tolerance

## Abstract

Silymarin (Sm) and dopamine (DA) act synergistically as potential antioxidants, mediating many physiological and biochemical processes. As a first report, we investigated the synergistic effect of Sm and DA in mitigating cadmium stress in *Phaseolus vulgaris* plants. Three experiments were conducted simultaneously using 40 cm diameter pots to elucidate how Sm and DA affect cadmium tolerance traits at morphological, physiological, and biochemical levels. Cadmium stress triggered a marked reduction in growth, productivity, and physio-biochemical characteristics of common bean plants compared to unstressed plants. Seed priming (SP) and foliar spraying (FS) with silymarin (Sm) or dopamine (DA) ((DA (SP) + Sm (FS) and Sm (SP) + DA (FS)) ameliorated the damaging effects of cadmium stress. Sm seed priming + DA foliar spraying (Sm (SP) + DA (FS)) was more efficient. The treated stressed common bean plants showed greater tolerance to cadmium stress by diminishing oxidative stress biomarkers (i.e., O_2_^•−^, H_2_O_2_, and MDA) levels through enhanced enzymatic (SOD, CAT, POD, APX) and non-enzymatic (ascorbic acid, glutathione, α-tocopherol, choline, phenolics, flavonoids) antioxidant activities and osmoprotectants (proline, glycine betaine, and soluble sugars) contents, as well as through improved photosynthetic efficiency (total chlorophyll and carotenoids contents, photochemical activity, and efficiencies of carboxylation (iCE) and PSII (*Fv/Fm*)), polyamines (Put, Spd, and Spm), and polyamine metabolic enzymes (ADC and ODC) accumulation. These findings signify that Sm and DA have remarkable anti-stress effects, which can help regulate plant self-defense systems, reflecting satisfactory plant growth and productivity. Thus, realizing the synergistic effect of Sm and DA in cadmium tolerance confers potential new capabilities for these compounds to function in sustainable agriculture.

## 1. Introduction

Vegetable crops are cultivated on a large scale all over the world due to their nutritional importance to humans and animals, including the common bean (*Phaseolus vulgaris* L.), whose seeds are rich in protein, dietary fiber, and carbohydrates [1]. It is an essential constituent of the agriculture sector worldwide (especially in developing countries). However, soils in dry regions require extensive mineral fertilizers application for plant nutrition, especially nitrogen (N) and phosphorus (P) fertilizers [2]. When fertilizers (particularly phosphorus (P)) are used extensively, cadmium ion (Cd^2+^) levels tend to increase in the soil in the soil, which are effortlessly absorbed and translocated to plant shoots [3,4].

Cadmium (Cd) is among the common heavy metal pollutants of plants, animals, and humans [5]. Cd contamination causes major disruption to the agricultural system because it effortlessly seeps from the soil into groundwater and thus poses a threat to human health and the environment [6]. In plants, cadmium disrupts the physiological processes (i.e., photosynthesis, transpiration, and respiration), reducing chlorophyll biosynthesis and damaging the light-harvesting complex and photosystems [7,8]. Accordingly, plants display different injury symptoms (e.g., leaf epinasty, chlorosis, and stunted growth). To alleviate Cd toxicity and peroxidation, plants have developed complex defense mechanisms via physiological adjustments and accumulation of secondary metabolites. Various enzymes and reducing metabolites (e.g., tocopherols, glutathione, ascorbate, etc.) are mechanisms that can tolerate ROS [6,9]. Recently, several practices of sustainable agronomic strategies have been recommended to improve plant self-defense systems in different agricultural crops to lessen the detrimental impacts of abiotic stresses. For instance, researchers recommend glycine betaine [10,11], glutathione [12,13,14,15], dopamine [16,17], silymarin [18,19], and others [20,21,22,23,24,25] for external use as anti-stressors.

Silymarin (Sm) is a bioactive chemical ingredient of the *Silybum marianum* plant. It is a flavonolignan and consists mainly of silybinin, silychristin, and silydianin (flavonoids) [19]. Sm has been exceedingly utilized as an anti-inflammatory, antiviral, and antioxidant chemical to protect the human liver and against various types of cancer [26,27,28]. Although Sm is known to possess diverse medicinal and biological activities in animals and humans [29,30,31], very little is known scientifically regarding its potential effects as a growth enhancer in major crops and obviously explains its effects on plants. Considering the multiple function of silymarin in animals and humans, studies have emerged to show that silymarin, as a potent antioxidant, can promote growth and productivity in stressed plants, reinforcing their defensive systems [18,19].

Dopamine (DA) is a water-soluble natural antioxidant, a catecholamine neurotransmitter in mammals, and it is present in several species of crop plants [16,17,32]. Dopamine was first explored in plants as a natural product possessing antioxidant strength comparable to that of ascorbate and gallocatechin gallate and greater than that of quercetin (flavonol), glutathione, catechin, and luteolin (flavone) [33]. Dopamine regulates plant sugar metabolism, active oxygen scavenging processes, and ion permeability. It also harmonizes plant hormones, affecting plant growth and productivity [32]. As it has reductive power, it plays essential functional roles in plant tolerance to abiotic stressors and in photophosphorylation of chloroplasts, ending in ROS removal [17]. Under salinity stress, exogenously applied DA regulates the aquaporin gene OsPIP1-3 expression in rice [34]. Drought-stressed potatoes treated with ultraviolet light or abscisic acid have a significantly increased DA content [35]. Dopamine also ameliorated damage in salt-stressed apples [36]. With exogenously applied DA, moderate drought-stressed apples displayed a noticeable increase in nutrient contents, absorption, and translocation [32].

At present, there are no reports on the importance and integration role of silymarin plus dopamine in alleviating cadmium stress. Thus, our study aimed at determining whether exogenously supplemented Sm and DA could attenuate cadmium stress in common bean. The combinations of seed priming + foliar spraying with DA + Sm or with Sm + DA, respectively, were compared for the highest efficacy in alleviating cadmium-stressed common bean. Our hypothesis was that an exogenous application of silymarin and dopamine would boost bean plant tolerance to cadmium stress by enhancing plant growth, modifying plant water status through elevating osmoprotectant and antioxidant contents, and improving enzymatic and non-enzymatic antioxidant activities. To test this, we assessed several physiological and biochemical parameters to explore the ability of common bean plant to tolerate cadmium stress conditions.

## 2. Materials and Methods

### 2.1. Growing Conditions of the Plant Material

*Phaseolus vulgaris* L. seeds (cultivar Bronco) were procured (Agricultural Research Center, Horticulture Research Institute, Egypt). After the surface of the seeds was sterilized with a 5% NaOCl solution and air-dried for 24 h, the seeds were subjected to pre-treatments (priming). The seeds were primed in distilled water, 250 μM silymarin (Sm), or 200 μM dopamine (DA) (both from Sigma-Aldrich, St Louis, MO, USA), based on treatment, for 2 h. The primed seeds were air-dried again for 24 h. Thereafter, using 120 pots (black plastic, diameter 40 cm, depth 38 cm), five seeds were sown in each pot. Each pot was filled with a suggested composite medium (ScM) [37]. The ScM consisted of crushed maize grains, vermiculite, peat moss, and compost in quantities of 15, 30, 50, and 5%, respectively. Humic acid and the fungicide Moncut SC (Central Glass Co., Ltd., Tokyo, Japan) were added in quantities of 250 and 125 mg L^−1^ to the ScM components. This medium (ScM) was enriched with the following fertilizer components: ammonium nitrate (415 mg L^−1^), calcium superphosphate (500 mg L^−1^), potassium sulfate (333 mg L^−1^), magnesium sulfate (833 mg L^−1^), iron (333 mg L^−1^), zinc (333 mg L^−1^), manganese (333 mg L^−1^), and CaCO_3_ (1250 mg L^−1^, to regulate the pH of SCM). The pots were harmoniously arranged in an open (net) greenhouse under natural eco-conditions, which were 27 ± 3 °C as mean temperature, 66 ± 5% as humidity, and 12/12 h as day/night photoperiods. Sixteen (16) days from sowing (DFS) (before the sixth watering on the same day), the seedlings were reduced to three per pot before the pots were split equally into two sets, each containing 60 pots (3 treatments × 4 replicates × 5 pots). The first set was divided into 3 treatments: (1) no stress (NS) + foliar spraying with distilled water + seed priming in distilled water (control); (2) NS + seed priming in DA + leafy treatment with Sm; and (3) NS + seed priming in Sm + leafy treatment with DA. In these treatments, plants were irrigated regularly every 3 days with cadmium (Cd)-free water. The second set was also divided into 3 treatments (4 replicates × 5 pots): (4) Cd stress (Cd-S) + foliar spraying with distilled water + seed priming in distilled water; (5) Cd-S + seed priming in DA + foliar spraying with Sm; and (6) Cd-S + seed priming in Sm + foliar spraying with DA. In the second set treatments, Cd-S was induced by adding 0.5 mM Cd (from Sigma-Aldrich, St. Louis, MO, USA) using CdSO_4_ in irrigation water starting from the 7th irrigation (19 DFS). Foliar spraying with Sm or DA was performed after 24 h from the 8th irrigation. The irrigation with Cd-containing water was continued until harvest, and the sprays were applied 3 times: 23, 32, 41 DFS.

The Cd (0.5 mM), Sm (250 μM), and DA (200 μM) concentrations as well as treatments (the combined seed + leafy treatments with Sm and DA outperformed the single treatments with either Sm or DA) were specified from the preliminary trial (Supplementary Table S1), as these concentrations and combinations of treatments gave the best responses and were therefore selected for the main study. The study was performed in triplicate, simultaneously, in the same period. The three trials were continued until harvest for green yield (56–65 DFS). However, at 45 DFS, the plant samples were collected to evaluate the parameters detailed below, except for green pod yield.

### 2.2. Evaluation of Growth and Yield Traits

At 45 DFS, 4 plants were randomly taken from each treatment. The leaf number was counted for each plant. Using graph sheets, the areas of all leaves on each plant were measured manually (all squares covered by leaves were counted as the leaf area). Then, the dry weight per plant was recorded after drying in an oven at 70 °C until constant weights were reached. At the stage of marketable green pods, all green pods on all remaining plants in each treatment were collected and weighed for pod number and pod weight per pot.

### 2.3. Evaluation of Photosynthetic Efficiency

The following determinations were made on the fully extended first and second upper leaves of each plant. The procedures of Konrad et al. [38] were utilized to evaluate the instantaneous carboxylation efficiency (iEC) (in µmol per m^2^ per s) and leafy contents of photosynthesis pigments (in mg per g FW), and those of Wellburn [39] were utilized to evaluate photochemical activity (µmol mg^−1^ chlorophyll min^−1^) utilizing the technique of KCN. The procedure in Ref [38] was applied to evaluate chlorophyll “a” fluorescence (Fv/Fm) using a fluorometer (PAM-2000, Heinz-Walz). 

### 2.4. Determination of Leafy Relative Content of Water (RWC) and Contents of Osmo-Regulatory Compounds

Leaf-blade discs with a diameter of 2 cm were prepared to determine the RWC [40]. Fresh weight (Fw), turgid weight (Tw), and dry weight (Dw) were recorded. FW was taken immediately after preparing the discs; TW was taken after the discs’ water saturation for 24 h; and DW was taken after drying for 48 h at 70 °C. These weights were then applied to RWC (%) = (Fw − Dw)/(Tw − Dw) × 100.

Free proline content (μmol per g leaf DW) was measured utilizing the method of Bates et al. [41]. The supernatant obtained from centrifugation of the extract was mixed with a solution of acid-ninhydrin (freshly prepared). After incubation for 0.5 h at 90°C, an ice-bath was used to terminate the reaction, and the extraction was performed again with toluene to discern both the aqueous and toluene phases. Free proline content was recorded after exposing the toluene phase to 520 nm and a standard curve. The procedure of Irigoyen et al. [42] was implemented to determine the total soluble sugars content (mg g^−1^ DW). The supernatant obtained from centrifugation of the extract was collected to mix with anthrone (freshly prepared). After incubation for 10 min at 100°C, the content of total soluble sugars was recorded after exposure to 625 nm and a standard curve. Both glycine betaine (GB) and choline contents were evaluated, applying the procedures of Subbarao et al. [43] and Bessieres et al. [44], respectively, using the HPLC system. The content of K^+^ (mg g^−1^ DW) was evaluated by applying the procedure of Bilal et al. [45]. Briefly, HNO_3_ and H_2_O_2_ were used for suspension and digestion of the samples, and ICP-MS (Optima 7900DV, Perkin-Elmer, Waltham, MA, USA) was utilized to quantify the content of K^+^.

### 2.5. Determination of Biomarkers of Oxidative Stress and Their Harmful Consequences for Cell Membranes

Hydrogen peroxide (H_2_O_2_) contents (molar extinction coefficient 0.28 µM^−1^ cm^−1^) were determined spectrophotometrically [46]. The plant leaf tissue (0.5 g) was extracted in acetone, and a titanium reagent and ammonium were added to the acetone extract and dissolved in sulfuric acid (1 M). The absorbance of the supernatant was measured at 415 nm, and the results of H_2_O_2_ contents were expressed as µmol g^−1^ FW.

To determine the concentration of O_2_^•−^, 100 mg of bean leaves was cut into 1 mm × 1 mm fragments and immersed for 1 h at room temperature in 10 mM K-phosphate buffer (pH 7.8), 0.05% NBT, and 10 mM NaN_3_. Then, 2 mL of the immersed solution was heated at 85°C for 15 min and rapidly cooled. Optical density was measured colorimetrically at 580 nm, and the content of O_2_^•−^ was expressed as A_580_ g^−1^ FW [47].

For the expression of lipid peroxidation, malondialdehyde (MDA) content (molar extinction coefficient at 155 mM^−1^ cm^−1^) was measured using the Heath and Packer [48] method. A 0.1 g sample of leaf tissue was homogenized with 5 mL of 0.07% NaH_2_PO_4_·2H_2_O and 1.6% Na_2_HPO_4_·12H_2_O (50 mM, pH 7.8). Under cooling (4 °C), the homogenized sample was centrifuged at 20,000 ×g for 25 min. The absorbance of the supernatant was read at 532 nm and corrected for non-specific turbidity at 600 nm. The observed units of MDA contents were expressed as µmol g^−1^ fresh weight (FW) [48].

To measure the leafy membrane stability index [49], two 0.2 g leafy blade samples were prepared in two tubes, each containing 10 mL of distilled water. The electrical conductivity of the solutions of the two samples (EC_a_ and EC_b_) were measured—the EC_a_ after heating for 0.5 h at 40 °C and the EC_b_ after boiling for 10 min at 100 °C—for application to MSI (%) = [1 − (EC_a_/EC_b_)] × 100.

Twenty leaf-blade discs were prepared to determine the electrolyte leakage (EL) [37]. The solution electrical conductivities EC_a_, EC_b_, and EC_c_ were recorded. EC_a_ was taken immediately after preparing the discs; EC_b_ was taken after the discs were heated for 0.5 h at 45–55 °C; and EC_c_ was taken after boiling for 10 min at 100 °C. These conductivities were then applied to EL (%) = [(EC_b_ − EC_a_)/EC_c_] × 100.

### 2.6. Evaluation of Cadmium (Cd), Silymarin (Sm), and Dopamine (DA)

Utilizing a Perkin-Elmer Model 3300-Atomic Absorption Spectrophotometer, the content of Cd (in mg kg^−1^ leaf DW) was evaluated [50]. Powdered dried samples (0.1 g) were subjected to 12 h digestion with a 1:5 acid mixture (80% perchloric acid + concentrated H_2_SO_4_). Then, distilled water was added to the digested samples until they reached 100 mL each. The diluted samples were then used to evaluate Cd^2+^ contents.

The procedures depicted in Refs [51,52] were implemented to evaluate the contents of silymarin (Sm). The leafy samples were extracted in a Soxhlet apparatus with 200 mL methanol, and the extracts were subjected to evaporation to dryness. The resulting dried samples were reconstituted in 25 mL HPLC-grade methanol. Utilizing methanol, the final samples were diluted (after reconstitution) to evaluate the contents (in µg per g DW) of Sm utilizing a Thermo Fisher Scientific HPLC (Waltham, MA, USA).

To quantify the endogenous DA content, the procedures outlined in Ref [53] were applied. The weight of leafy tissue (100 mg) was ground and extracted with 1.5 mL 90% methanol. Then, the extracts were subjected to 10 min ultrasonic crushing. The extracts were then subjected to 10 min centrifugation under cooling (4 °C) at 12,000 rpm. After the resulting supernatants were filtered through a water filter membrane (0.22 µm), the filtrates were tested by QTRAP5500 LC–MS (AB Sciex Pret. Ltd., Framingham, MA, USA) utilizing a mobile phase containing NH_4_HCO_2_ (ammonium formate), C_2_H_3_N (acetonitrile), and H_2_O. The flow rate was 0.3 mL min^−1^, and 5 μL was used as a sample injection volume.

### 2.7. Determination of Non-Enzymatic Antioxidant Activities 

The detailed procedures described in refs [54,55] were applied for assessing the contents (µmol g^−1^ FW) and the redox status (%) of both ascorbic acid (AsA) and glutathione (GSH), respectively. The antioxidant α-tocopherol was determined for its content (in μmol per g DW) utilizing the HPLC system [56,57]. The total leaf content of phenols was evaluated utilizing the Folin–Ciocalteu procedure described in Ref [58], with some modifications [59]. The content of total flavonoids was evaluated utilizing the procedure of Sultana et al. [60]. To evaluate the iodine (I) content, dried leafy samples of common bean were prepared for assaying. The dried samples were incubated with tetramethylammonium hydroxide (TMAH; 25%) before applying the assaying standard method (prEN 15111: R2-P5-F01., 2006).

### 2.8. Evaluation of Antioxidant Enzymes’ Activity

The procedure detailed in Ref [61] was applied to measure superoxide dismutase (SOD) activity by observing the readings of NBT photochemical reduction inhibition at 560 nm. The enzyme amount inhibiting 50% of the NBT reduction was calculated as 1 SOD unit. The procedure of guaiacol oxidation [62] was applied to estimate the activity of peroxidase (POD) using an enzyme extract and phosphate buffer (100 mM, pH 7.5) and guaiacol and H_2_O_2_ as reaction substrates. The full procedure of Chandlee and Scandalios [63] with a modification by Ali et al. [59] was applied to evaluate catalase (CAT) activity using an enzyme extract and phosphate buffer (100 mM, pH 7.5) and H_2_O_2_ as a reaction substrate. The procedure of Asada and Takahashi [64] was applied to assess the activity of ascorbate peroxidase (APX) using an enzyme extract and phosphate buffer (100 mM, pH 7.5) and ascorbate as a reaction substrate.

### 2.9. Evaluation of Polyamine Contents

To evaluate the endogenous free polyamines (fPAs) contents (e.g., spermine, spermidine, and putrescine) in this study, the procedures of Kotzabasis et al. [65] were applied with a minor modification. The weights of leafy samples, 0.3 g each, were finely powdered in liquid N. All samples were then each homogenized in 3 mL of pre-cooled HClO_4_ (5%, v/v). The homogenates were subjected to 1 h incubation and 30 min centrifugation at 30,600 ×g both at 4 °C. All supernatants of all samples were utilized to assess fPAs by mixing 1 mL supernatant with 1 mL NaOH (2 M) + 10 μL C_7_H_5_ClO (benzoyl chloride) in a tube. After vortexing the mixture for 20s, a 30 min incubation was performed at 37 °C. After adding the reaction mixture + 2 mL solution of saturated NaCl, the benzoyl fPAs were extracted with 2 mL of (C_2_H_5_)_2_O. The samples were subjected to 5 min centrifugation at 2300 ×g. The ether phase (1 mL) was obtained for drying, which was dissolved again in 500 μL CH_3_OH (60%, *v*/*v*) and then filtered. The contents of fPAs were evaluated utilizing a HPLC system with a mobile phase containing 58 C_2_H_5_OH: 2.5 C_2_H_3_N: 39.5 H_2_O (*v*/*v*/*v*) at 1 mL per min flow rate. Similar to the leafy samples, the standard curves for the fPAs were produced.

### 2.10. Assaying for Polyamine Biosynthetic Enzymes and Polyamine Oxidase (PAO) Activities

The activities of arginine and ornithine decarboxylases (ADC and ODC), respectively, were determined [66], with a minor modification. Leafy samples (0.5 g each) were homogenized in 100 mM P-buffer (pH 6.3) containing 5 mM EDTA + 1 mM C_8_H_10_NO_6_P (pyridoxal phosphate) + 10 mM C_4_H_10_O_2_S_2_ (dithiothreitol) + 25 mM C_6_H_8_O_6_ (ascorbic acid) + 0.1% (C_6_H_9_NO)n (polyvinylpyrrolidone). The homogenates were subjected to 1 h incubation and 20 min centrifugation at 9200× *g* at 4 °C for both. The supernatants were utilized to assay the enzyme activities. ADC and ODC activities were assayed utilizing a reaction mixture: 100 mM Tris–HCl buffer (pH 7.5) containing 5 mM EDTA + 40 μM C_8_H_10_NO_6_P (PLP) + enzyme extract (0.8 mL). The mixture was subjected to 2 min incubation at 37 °C utilizing a water bath. Thereafter, 25 mM L-Arginine (0.2 mL, pH 7.5) or 25 mM L-Ornithine (0.2 mL) was added. A 1 h incubation was performed at 37 °C; then, PCA was added (it was added before the 1 h incubation for controls) until it reached 5%. Then, the mixture was subjected to 5 min centrifugation at 2300× *g* under cooling (4 °C). A 0.5 mL supernatant was gathered to mix with 1 mL NaOH (2 mM) and 10 μL C_7_H_5_ClO (benzoyl chloride). Then, the mixture was subjected to 20 s vortexing and 12 h incubation at 37 °C. The reaction mixture was added to a 2 mL solution of saturated NaCl; then, the benzoyl fPAs were extracted with 2 mL (C_2_H_5_)_2_O, and the extracts were subjected to 5 min centrifugation at 2300× *g*. Then, the ether phase (1 mL) was obtained for drying, which was dissolved in 3 mL CH_3_OH (60%). The absorption of the CH_3_OH solution was recorded at 254 nm.

Polyamine oxidase (PAO) activity was determined by implementing the procedures depicted in Refs [67,68], with a minor modification. Leafy samples (300 mg) were homogenized, with each sample subjected to 0.1 M P-buffer (pH 6.5) under cooling (4 °C). The homogenates were subjected to 20 min centrifugation at 9200× *g* under cooling, and the resulting supernatants were utilized to assay the enzyme activities. The reaction mixture consisted of 2.5 mL P-buffer (0.1 M) + 0.2 mL 4-amionoantipyrine/N,N-dimethylaniline + 0.1 mL horse-radish peroxidase (250 unit per mL) + 0.4 mL enzyme extract. Spermidine (up to 20 mM) was added to initiate the assay for PAO evaluation. PAO activity was assayed by computing a change of 0.001 absorbance unit in OD at 550 nm.

### 2.11. Statistical Analysis

The treatments were deposited into a complete random block design, and the resulting data were analyzed using one-way ANOVA after testing for homogeneity of error variances following the Gomez and Gomez [69] procedures. A combined analysis of data from three trials was carried out, and significant differences among and between treatments were compared at *p* ≤ 0.05 by Duncan’s multiple range test. Pearson’s correlation coefficients, heatmap, and PCA biplot graph were performed using R software (version 4.1.3, https://CRAN.R-project.org, accessed on 25 September 2022).

## 3. Results

A preliminary study was conducted to identify the most efficient concentrations, enhancing growth and productivity, of dopamine (DA) and silymarin (Sm) applied as seed soaking and foliar spraying on *Phaseolus vulgaris* plants, as well as to identify Cd concentrations that negatively affect plant growth and productivity (Supplementary Table S1). The selected treatment combinations were dependent on the variables tested, i.e., leaf number and area, plant dry weight, pod number, pod yield, and total chlorophyll. Different combinations of seed priming (SP) and foliar spraying (FS) with silymarin (Sm) or dopamine (DA) ((DA (SP) + Sm (FS) and Sm (SP) + DA (FS)) showed the most efficient results (Supplementary Table S1), and consequently, these treatments were selected for the main study.

The data in Table 1 show the impact of seed and leafy treatments on the contents of Sm and DA. They were notably boosted in cadmium-stressed plants by 42.3% and 107.1%, respectively, when compared to the control-grown plants. Cadmium-stressed bean plants were able to accumulate more silymarin and dopamine upon receiving Sm or DA exogenous application (DA (SP) + Sm (FS)) or (Sm (SP) + DA (FS)). Silymarin seed priming + dopamine foliar spraying (Sm (SP) + DA (FS)) was more efficient, as Sm and DA were increased by 79.7% and 68.9%, respectively, as compared with cadmium-stressed grown plants.

**Figure 1:**
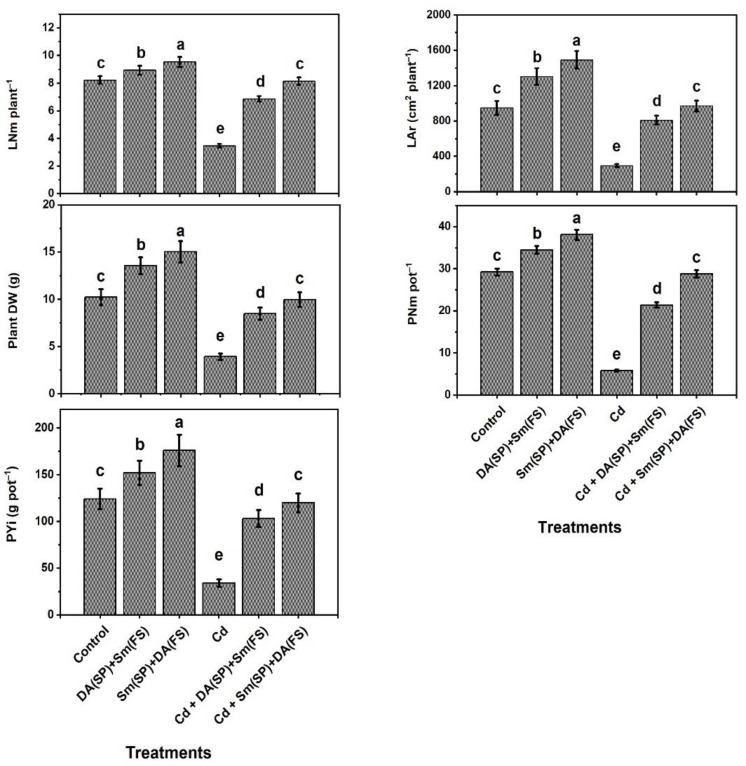
Effects of seed priming (SP) and foliar spraying (FS) with silymarin (Sm) or dopamine (DA) on growth and yield characteristics of cadmium-stressed *Phaseolus vulgaris* plants. Columns labeled with same or different letters within each plot signify non-significant or significant differences, respectively, based on LSD test (*p* ≤ 0.05). Bars represent SE values (three replicates). LNm = leaf number, LAr = leaf area, DW = dry weight, PNm = pod number, and PYi = pod yield.

Cadmium-stressed bean plants (no Sm or DA exogenous application) showed symptoms of Cd damage (e.g., necrosis, chlorosis, and stunted growth). Moreover, a marked decrease in leaf number (57.9%), leaf area (69.1%), DW (61.6%), pod number (80.1%), and pod yield (72.6%) was noticed in bean plants subjected to cadmium stress relative to the control-grown plants (Figure 1). Sm or DA exogenous application (DA (SP) + Sm (FS)) or (Sm (SP) + DA (FS)) significantly enhanced the growth (leaf number, leaf area, and DW) and yield (pod number and pod yield) characteristics in both cadmium-stressed and unstressed plants. Silymarin seed priming + dopamine foliar spraying (Sm (SP) + DA (FS)) was more effective, as all the growth traits were increased by 135.7%, 229.3%, 154.6%, 396.6%, and 252.9%, respectively, as compared to the cadmium-stressed grown plants (Figure 1).

**Figure 2:**
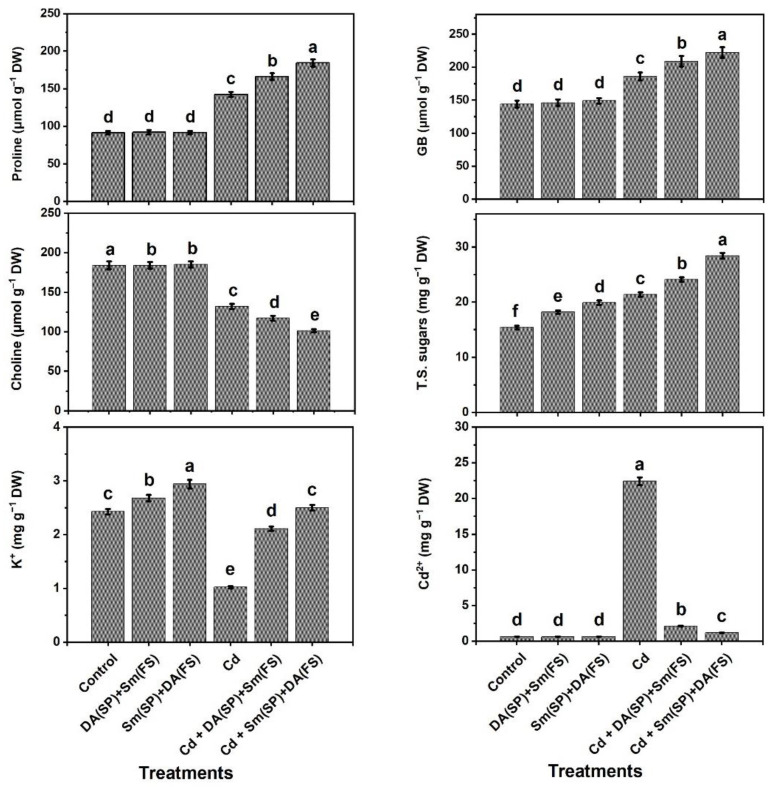
Effects of seed priming (SP) and foliar spraying (FS) with silymarin (Sm) or dopamine (DA) on osmoprotectants and cadmium contents of cadmium-stressed *Phaseolus vulgaris* plants. Columns labeled with same or different letters within each plot signify non-significant or significant differences, respectively, based on LSD test (*p* ≤ 0.05). Bars represent SE values (three replicates). GB = Glycine betaine, T.S. sugars = Total soluble sugars, K^+^ = Potassium ion, and Cd^2+^ = Cadmium ion.

The data in Figure 2 represent the contents of osmoprotectants in terms of proline, glycine betaine, and total soluble sugars, which increased significantly in cadmium-stressed plants by 55.8%, 26.16%, and 38.9%, respectively, compared to the control-grown plants. However, Sm or DA exogenous application (DA (SP) + Sm (FS)) or (Sm (SP) + DA (FS)) increased the above-mentioned osmoprotectants in cadmium-stressed plants. In contrast, the choline contents of cadmium-stressed bean plants were significantly decreased by 28.3% compared with the control-grown plants. Under cadmium stress conditions, the exogenous application of Sm or DA substantially increased the contents of the osmoprotectants tested (proline, glycine betaine, total soluble sugars) in comparison with un-treated plants and noticeably exceeded those obtained under normal conditions, whereas choline contents were substantially decreased under cadmium stress conditions (Figure 2). The content of Cd^2+^ ion was noticeably elevated under cadmium stress conditions compared with the control-grown plants. However, Sm or DA exogenous application (DA (SP) + Sm (FS)) or (Sm (SP) + DA (FS)) significantly reduced Cd^2+^ ion accumulation by 90.6% and 94.1%, respectively, compared to cadmium-stressed plants (Figure 2).

**Figure 3:**
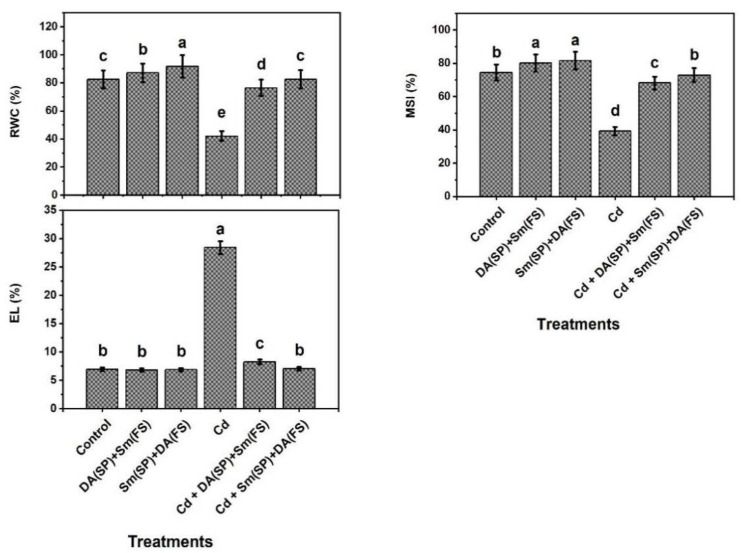
Effects of seed priming (SP) and foliar spraying (FS) with silymarin (Sm) or dopamine (DA) on plant water status of cadmium-stressed *Phaseolus vulgaris* plants. Columns labeled with same or different letters within each plot signify non-significant or significant differences, respectively, based on LSD test (*p* ≤ 0.05). Bars represent SE values (three replicates). RWC = relative water content, MSI = membrane stability index, and EL = electrolyte leakage.

Plant water status was measured as relative content of water (RWC %), stability index of membranes (MSI %), and leakage of ions (EL %) (Figure 3). Under cadmium stress conditions, the RWC and MSI were noticeably decreased by 48.9 and 47.2 %, while the EL was increased by 309.5 %. Both Sm or DA exogenous application (DA (SP) + Sm (FS)) and (Sm (SP) + DA (FS)) considerably raised the RWC and MSI and also considerably diminished the EL in cadmium-stressed and control plants. In comparison with cadmium-stressed plants, the RWC and MSI were significantly increased with (DA (SP) + Sm (FS)) or (Sm (SP) + DA (FS)) exogenous application by (81.5 and 96.2 %) and (73.5 and 85.5 %), respectively, whereas Sm (SP) + DA (FS) was more efficient. While electrolyte leakage was increased in cadmium-exposed plants, (DA (SP) + Sm (FS)) or (Sm (SP) + DA (FS)) exogenous application improved plant water status to be approximately the same as that in control plants (Figure 3).

**Figure 4:**
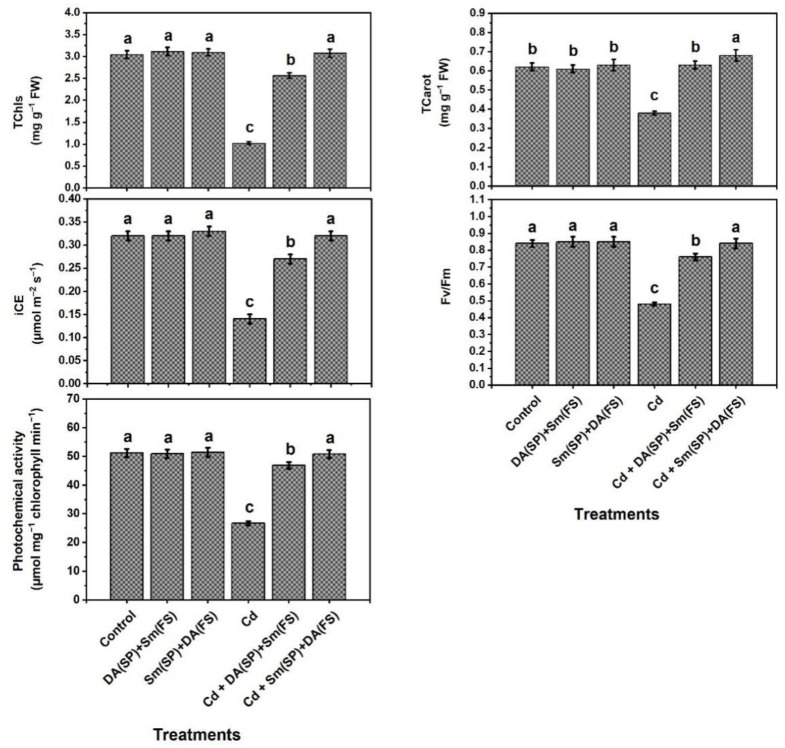
Effects of seed priming (SP) and foliar spraying (FS) with silymarin (Sm) or dopamine (DA) on photosynthesis efficiency of cadmium-stressed *Phaseolus vulgaris* plants. Columns labeled with same or different letters within each plot signify non-significant or significant differences, respectively, based on LSD test (*p* ≤ 0.05). Bars represent SE values (three replicates). TChls = Total chlorophylls, TCarot = Total Carotenoids, iCE = instantaneous carboxylation efficiency, and Fv/Fm = chlorophyll “a” fluorescence.

Under cadmium stress conditions, the photosynthetic efficiency indicators (in terms of leaf total chlorophyll and carotenoid contents, photochemical activity, and efficiencies of carboxylation (iCE) and PSII (*Fv/Fm*)) were markedly decreased by 66.4, 38.7, 47.8, 56.25, and 42.8%, respectively, relative to the control-grown plants (Figure 4). Sm or DA exogenous application (DA (SP) + Sm (FS)) or (Sm (SP) + DA (FS)) significantly enhanced plant photosynthetic efficiency in cadmium-stressed plants. Silymarin seed priming + dopamine foliar spraying (Sm (SP) + DA (FS)) was more effective, as all the mentioned traits were raised by 155.8, 78.9, 90.3, 128.6, and 75.1%, respectively, as compared to the cadmium-stressed grown plants (Figure 4).

**Figure 5:**
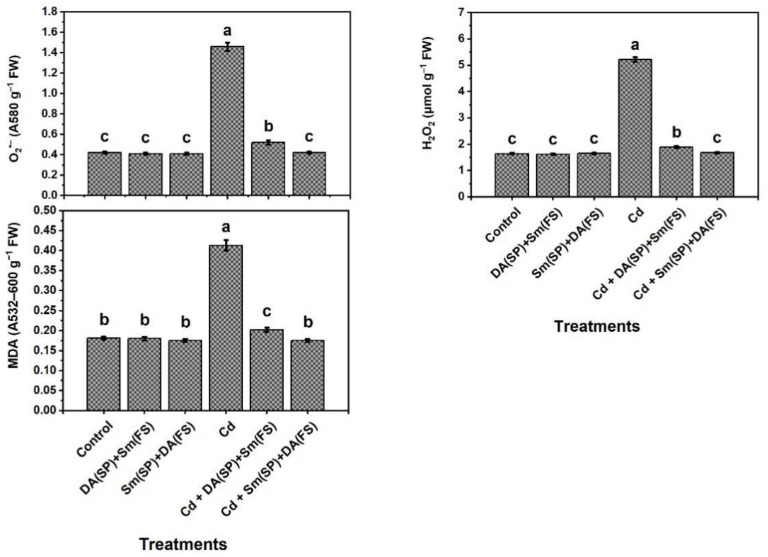
Effects of seed priming (SP) and foliar spraying (FS) with silymarin (Sm) or dopamine (DA) on stress marker levels of cadmium-stressed *Phaseolus vulgaris* plants. Columns labeled with same or different letters within each plot signify non-significant or significant differences, respectively, based on LSD test (*p* ≤ 0.05). Bars represent SE values (three replicates). O_2_^•−^ = Superoxide radical, H_2_O_2_ = Hydrogen peroxide, and MDA = Malondialdehyde.

Elevated levels of O_2_^•−^, H_2_O_2_, and MDA were observed in cadmium-stressed plants (Figure 5). The increases were 247.6%, 217.7%, and 128.2%, respectively, relative to the control-grown plants. In cadmium stress conditions, O_2_^•−^, H_2_O_2_, and MDA were significantly decreased with Sm or DA exogenous application (DA (SP) + Sm (FS)) or (Sm (SP) + DA (FS)), whereas (Sm (SP) + DA (FS)) was more efficient. The decreases were 71.2%, 67.8%, and 57.6%, respectively, compared to cadmium-stressed plants.

**Figure 6:**
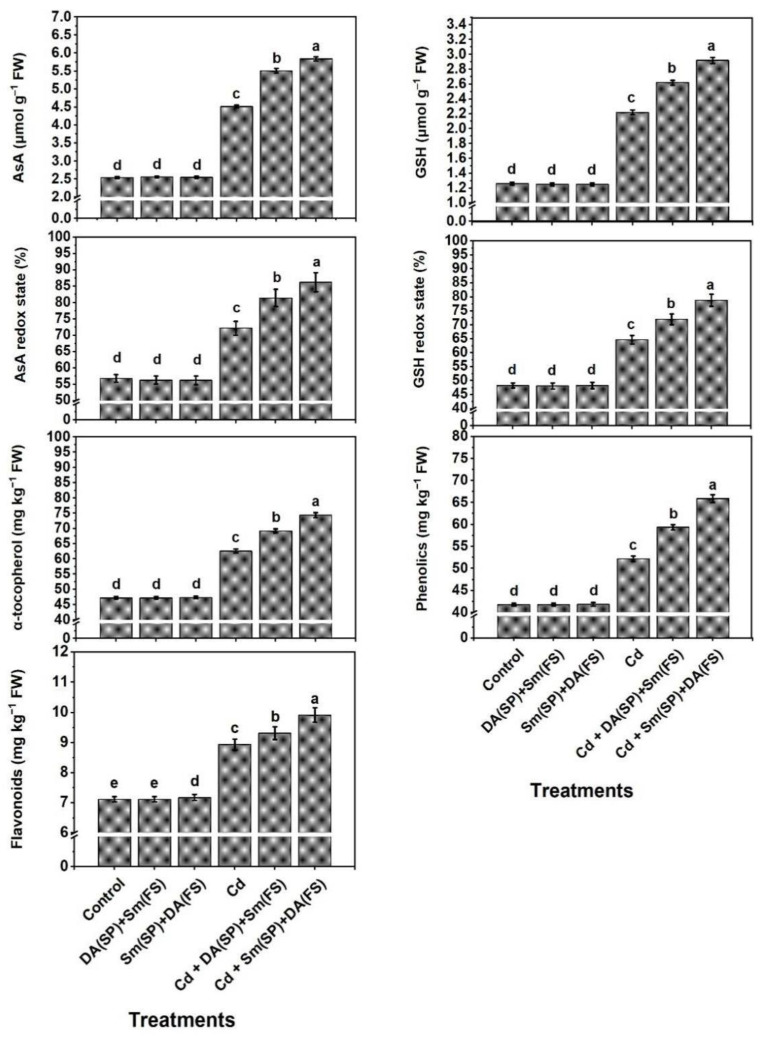
Effects of seed priming (SP) and foliar spraying (FS) with silymarin (Sm) or dopamine (DA) on non-enzymatic antioxidant levels of cadmium-stressed *Phaseolus vulgaris* plants. Columns labeled with same or different letters within each plot signify non-significant or significant differences, respectively, based on LSD test (*p* ≤ 0.05). Bars represent SE values (three replicates). AsA = Ascorbate, GSH = Glutathione.

The data in Figure 6 represent non-enzymatic antioxidant contents in terms of AsA, GSH, their states of redox, which increased significantly in cadmium-stressed plants by 77.9%, 76.2%, 27.1%, and 33.9%, respectively, as well as the contents of α-tocopherol, phenolic, and flavonoids, which were also increased by 32.6%, 24.9%, and 25.6%, respectively, when compared to the control-grown plants. Cadmium-stressed bean plants could accumulate more antioxidant (e.g., AsA, GSH, α-tocopherol, phenolic, and flavonoids) levels with power redox to cope with oxidative stress markers (O_2_^•−^, H_2_O_2_, and MDA) upon receiving Sm or DA exogenous application (DA (SP) + Sm (FS)) or (Sm (SP) + DA (FS)). Silymarin seed priming + dopamine foliar spraying (Sm (SP) + DA (FS)) was more effective, as all the mentioned traits were raised by 29.2%, 31.5%, 19.4%, 21.8%, 18.8%, 26.2%, and 10.9%, respectively, as compared to the cadmium-stressed grown plants (Figure 6).

**Figure 7:**
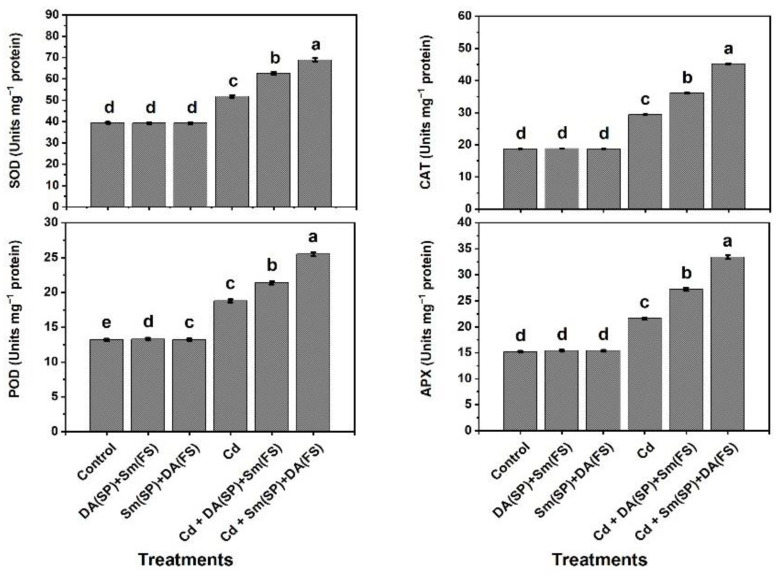
Effects of seed priming (SP) and foliar spraying (FS) with silymarin (Sm) or dopamine (DA) on enzymatic antioxidant activities of cadmium-stressed *Phaseolus vulgaris* plants. Columns labeled with same or different letters within each plot signify non-significant or significant differences, respectively, based on LSD test (*p* ≤ 0.05). Bars represent SE values (three replicates). SOD = Superoxide dismutase, CAT = Catalase, POD = Peroxidase, APX = Ascorbate peroxidase.

Under cadmium stress conditions, the enzymatic antioxidant activities in terms of SOD, CAT, POD, and APX were significantly raised by 31.5%, 57.2%, 42.4%, and 42.1%, respectively, relative to the control-grown plants (Figure 7). Upon receiving Sm or DA exogenous application (DA (SP) + Sm (FS)) or (Sm (SP) + DA (FS)), cadmium-stressed bean plants could maximize the enzyme activities with a significant preference for silymarin seed priming + dopamine foliar spraying (Sm (SP) + DA (FS)), increasing the activities by 33.1%, 53.7%, 35.6%, and 54.6%, respectively (Figure 7).

**Figure 8:**
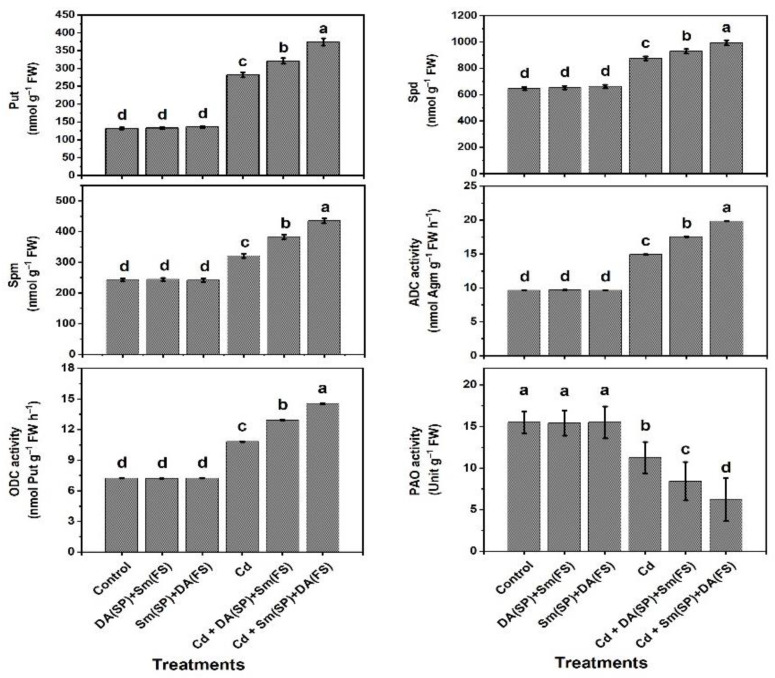
Effects of seed priming (SP) and foliar spraying (FS) with silymarin (Sm) or dopamine (DA) on polyamine content (Put, Spd, and Spm) and activities of polyamine metabolic enzymes (ADC, ODC, and PAO) of cadmium-stressed *Phaseolus vulgaris* plants. Columns labeled with same or different letters within each plot signify non-significant or significant differences, respectively, based on LSD test (*p* ≤ 0.05). Bars represent SE values (three replicates). Put = putrescine, Spd = Spermidine, Spm = Spermine, ADC = Arginine decarboxylase, ODC = Ornithine decarboxylase, and PAO = Polyamine oxidase.

The contents of polyamines in terms of putrescine (Put), spermidine (Spd), and spermine (Spm), in addition to the polyamine metabolizing enzyme (ADC and ODC) activities, were significantly elevated under cadmium stress conditions by 113.6%, 35.5%, 32.6%, 54.8%, and 49.4%, respectively, while the level of PAO was markedly decreased by 27.5% relative to the control-grown plants (Figure 8). Both Sm or DA exogenous application (DA (SP) + Sm (FS)) and (Sm (SP) + DA (FS)) considerably increased Put, Spd, Spm, ADC, and ODC and significantly decreased PAO under cadmium-stress conditions, with a significant preference for silymarin seed priming + dopamine foliar spraying (Sm (SP) + DA (FS)), which increased Put, Spd, Spm, ADC, and ODC by 32.6%, 13.6%, 35.5%, 33.1%, and 34.6%, respectively, while decreasing the levels of PAO by 26.2% compared to untreated plants (Figure 8).

**Figure 9:**
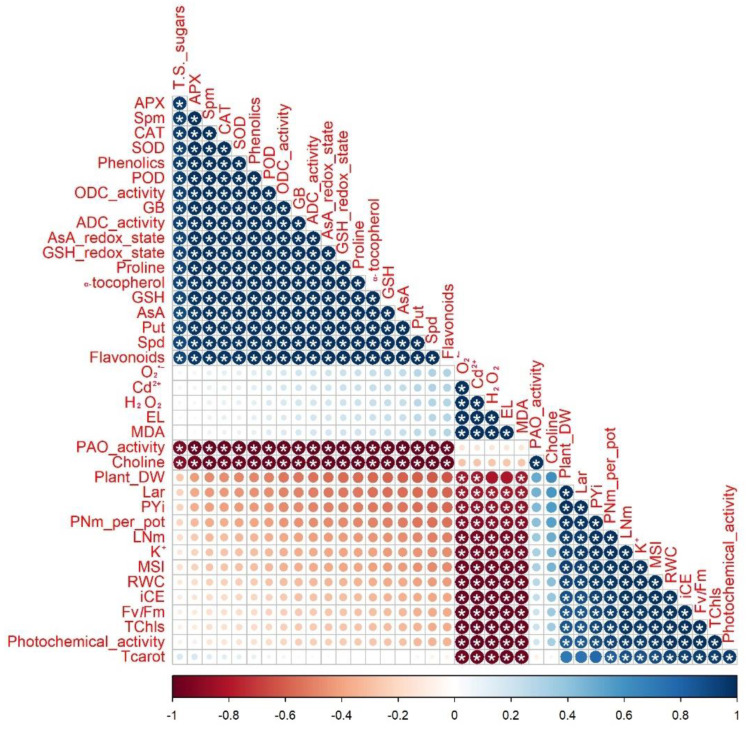
Graph of Pearson’s correlation analysis among the parameters examined. The colors represent variations in the resulting data. * indicates significance at *p* ≤ 0.05. TChls = Total chlorophyll, TCarot = Total Carotenoids, iCE = instantaneous carboxylation efficiency, Fv/Fm = chlorophyll “a” fluorescence, LNm = leaf number, LAr = leaf area, DW = dry weight, PNm = pod number, PYi = pod yield, AsA = Ascorbate, GSH= Glutathione, O_2_^•−^ = Superoxide, H_2_O_2_ = Hydrogen peroxide, MDA = Malondialdehyde, EL = Electrolyte leakage, RWC = Relative water content, SOD = Superoxide dismutase, CAT= Catalase, APX = Ascorbate peroxidase, POD = Peroxidase, Put = putrescine, Spd = Spermidine, Spm = Spermine, ADC = Arginine decarboxylase, ODC = Ornithine decarboxylase, and PAO = Polyamine oxidase.

The analysis of Pearson’s correlation was generated to detect the relationship between the parameters obtained in cadmium-stressed *Phaseolus vulgaris* plants treated with silymarin (Sm) or dopamine (DA) as seed priming (SP) and/or foliar spraying (FS) (Figure 9). The findings displayed a noticeably positive correlation (*p* ≤ 0.05) between plant DW, Lar, PYi, PNm per pot, and LNm and the levels of K^+^, MSI, RWC, iCE, Fv/Fm, Tchls, and photochemical activity. Meanwhile, they were negatively correlated (*p* ≤ 0.05) with MDA, EL, H_2_O_2_, O_2_^•−^, and Cd^2+^ levels. Moreover, the activities of APX CAT, SOD, POD, ODC, ADC ASA redox, and GSH redox and the levels of T. S. sugars, Spm, phenolics, GB, ASA, GSH, proline, Put, Spd, and α-tocopherol were significantly positively (*p* ≤ 0.05) correlated with each other, while, interestingly, they were significantly negatively correlated (*p* ≤ 0.05) with PAO activity and choline level (Figure 9).

**Figure 10:**
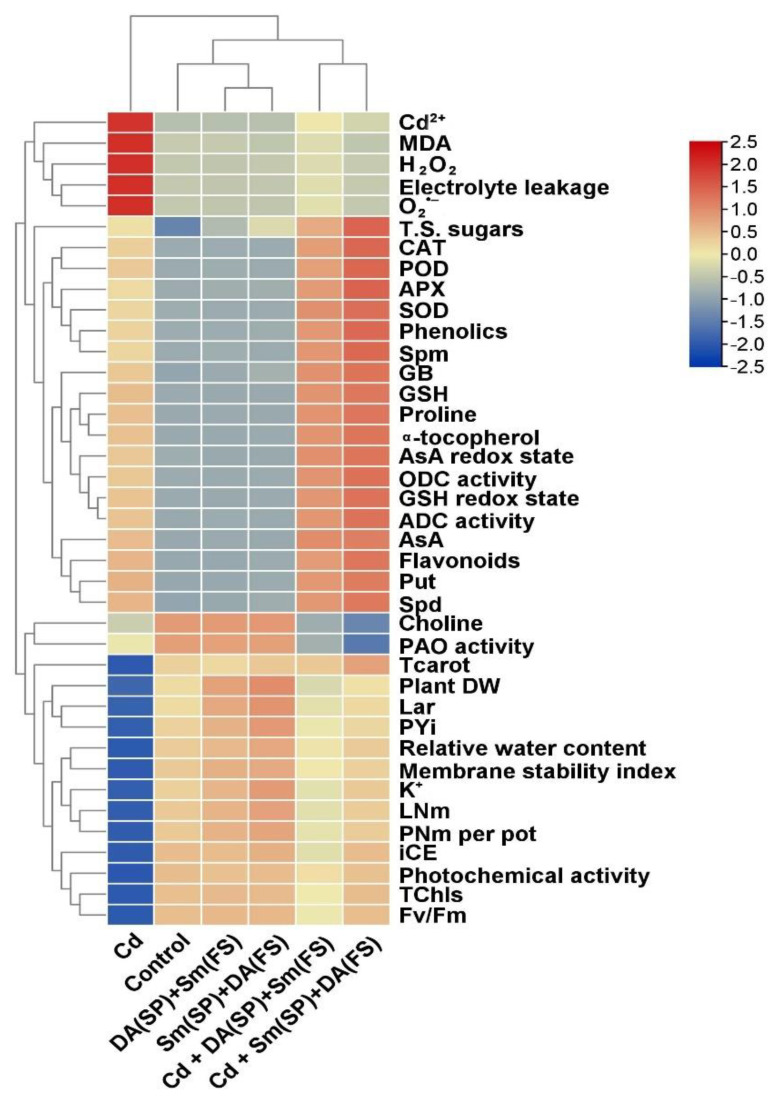
The heatmap graph shows analysis of hierarchical clustering between the examined parameters and treatments. The colors represent variations in the resulting data. TChls = Total chlorophyll, TCarot = Total Carotenoids, iCE = instantaneous carboxylation efficiency, Fv/Fm = chlorophyll “a” fluorescence, LNm = leaf number, LAr = leaf area, DW = dry weight, PNm = pod number, PYi = pod yield, AsA = Ascorbate, GSH = Glutathione, O_2_^•−^ = Superoxide, H_2_O_2_ = Hydrogen peroxide, MDA = Malondialdehyde, EL = Electrolyte leakage, RWC = Relative water content, SOD = Superoxide dismutase, CAT = Catalase, APX = Ascorbate peroxidase, POD = Peroxidase, Put = putrescine, Spd = Spermidine, Spm = Spermine, ADC = Arginine decarboxylase, ODC = Ornithine decarboxylase, and PAO = Polyamine oxidase.

In addition to the correlation analysis, the relation between the parameters studied and the interactive treatments with silymarin (Sm) or dopamine (DA) exogenous application as seed priming (SP) and/or foliar spraying (FS) for cadmium-stressed *Phaseolus vulgaris* plants was examined using the heatmap with hierarchical analysis. The different treatments were divided into three main groups based on the hierarchical cluster. The treatment with Cd stress alone was clustered in the first group, while treatments with DA (SP) + Sm (FS), Sm (SP) + DA (FS), and the control were clustered in the second group, which performed better than Cd treatment. The treatments with Cd + DA (SP) + Sm (FS) and Cd + Sm (SP) + DA (FS) were clustered in the third group, which also performed better than Cd treatment alone (first group). The treatments in the third group effectively improved the levels of GSH, T.S. sugars, phenolics, Spm, GB, proline, α-tocopherol, AsA, Put, Spd, flavonoids, as well as enhancing the activity of CAT, APX, POD, and SOD and improving the redox status of AsA and GSH as compared with those in the first group. Moreover, they decreased the levels of Cd, MDA, H_2_O_2_, EL, and O_2_^•−^ while improving the plant DW, Lar, PYi, RWC, MSI, K, LNm, iCE, photochemical activity TCHs, and Fv/Fm as compared with the first group. Therefore, the results obtained indicated that seed priming and foliar spraying with Sm and DA alleviated the negative impacts of Cd stress in *Phaseolus vulgaris* plants, as well as enhancing the growth and physiological and biochemical properties of Cd-stressed plants (Figure 10).

**Figure 11:**
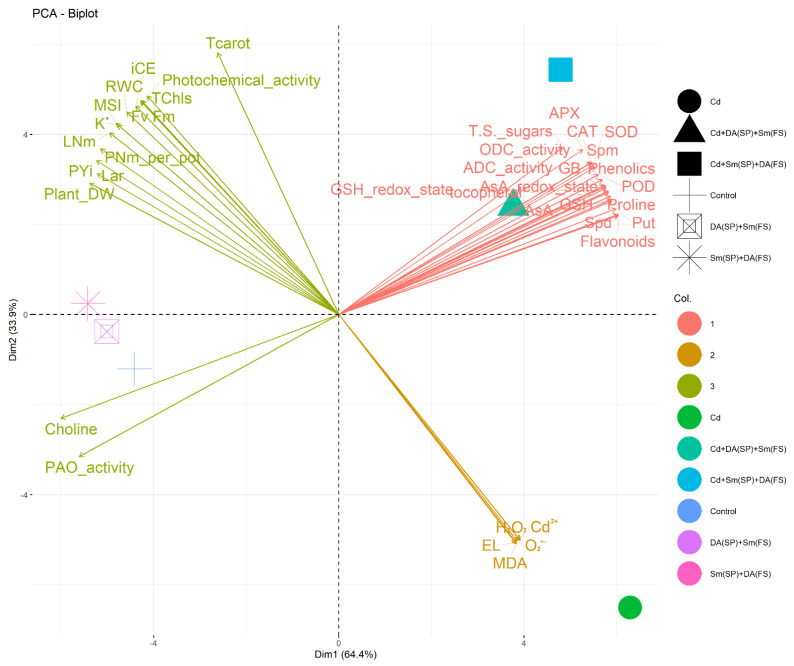
Biplot graph of different parameters studied for each treatment, showing the first two dimensions (Dim1 and Dim2) of the principal component analysis (PCA) model in *Phaseolus vulgaris* plants treated with silymarin (Sm) and dopamine (DA) as seed priming (SP) and foliar spraying (FS) under cadmium stress conditions. TChls = Total chlorophyll, TCarot = Total Carotenoids, iCE = instantaneous carboxylation efficiency, Fv/Fm = chlorophyll “a” fluorescence, LNm = leaf number, LAr = leaf area, DW = dry weight, PNm = pod number, PYi = pod yield, AsA = Ascorbate, GSH = Glutathione, O_2_^•−^ = Superoxide, H_2_O_2_ = Hydrogen peroxide, MDA = Malondialdehyde, EL = Electrolyte leakage, RWC = Relative water content, SOD = Superoxide dismutase, CAT = Catalase, APX = Ascorbate peroxidase, POD = Peroxidase, Put = putrescine, Spd = Spermidine, Spm = Spermine, ADC = Arginine decarboxylase, ODC = Ornithine decarboxylase, and PAO = Polyamine oxidase.

Owing to the high variation resulting from seed priming and foliar spraying of *Phaseolus vulgaris* plants with Sm and DA under Cd stress treatments, we performed a biplot of the principal component analysis (PCA) to display the impact of treatments on all the studied parameters. The two components of PCA dimensions (Dim1 and Dim2) indicated 64.4% and 33.9% of data variability, respectively, under the experimental conditions (Figure 11). Under Cd stress, DA and Sm application as seed priming and foliar spraying enhanced the SOD, CAT, APX, POD ADC, and ODC activities, levels of AsA, GSH, α-tocopherol, Fv/Fm, proline, Spd, Put, total soluble sugars, phenolics, ASA redox, GSH redox state, while Cd^2+^ contents, MDA, H_2_O_2_, EL, and O_2_ were depressed. Under non-Cd stress conditions, DA and Sm application as seed priming and foliar spraying enhanced the RWC, iCE, Tcarot, photochemical activity, MSI, TClls, K^+^, LNm, PNm, PYi, Lar, and plant DW (Figure 11). Therefore, DA and Sm exogenous application has a very important role in improving the growth and biomass production of *Phaseolus vulgaris* plants under non-Cd and Cd stress conditions.

## 4. Discussion

In this investigation, common bean plants were grown under cadmium stress conditions, resulting in many morphological, physiological, and biochemical disorders. Common bean plants reacted to cadmium stress by decreasing growth and productivity (Figure 1), total chlorophyll and carotenoids contents, photochemical activity, and efficiencies of carboxylation (iCE) and PSII (*Fv/Fm*) (Figure 4), relative water content (RWC) and membrane stability index (MSI) (Figure 3), and the content of K^+^ and activity of polyamine oxidase (PAO) (Figure 2 and Figure 8) relative to the control-grown plants. Contrarily, non-enzymatic and enzymatic antioxidant activities, osmoprotectant (except for choline) contents, as well as Sm and DA contents were improved due to the increased oxidative stress and its consequences (O_2_^•−^, H_2_O_2_, MDA, and EL) and Cd^2+^ levels (Figure 2, Figure 3, Figure 4, Figure 5, Figure 6 and Figure 7). 

The disturbance of physiological and biochemical processes (i.e., photosynthesis, transpiration, and respiration), reducing chlorophyll, protein, and nucleic acid biosynthesis due to the increased level of ROS, results in different symptoms of damage (e.g., leaf epinasty, chlorosis, and stunted growth), leading to aging and death in crops [4,70,71]. Higher levels of ROS (O_2_^•−^ and H_2_O_2_), MDA, and EL are regarded as oxidative stress biomarkers. During the current study, Cd stress conditions enhanced these biomarkers in bean plants. This connection between oxidative stress and heavy metals has been reported by many investigators [72,73]. To cope with Cd toxicity and the redox status, plants have developed complex cellular defense mechanisms via physiological adjustments and the accumulation of secondary metabolites in which protective enzymes (POD, CAT, APX, and SOD) and reducing metabolites (AsA, glutathione, and tocopherol flavonoids) are implicated [6,74]. Osmo-regulators, such as proline, glycine betaine (GB), and soluble sugars, are also associated with the maintenance of cellular integrity of plants to ameliorate oxidative stress [18,74,75]. However, endogenous antioxidants are often not enough to ameliorate the adverse effects of abiotic stressors. Several procedures have been demonstrated to exogenously immunize plants with protective (anti-stress) substances against environmental stresses, including foliar spraying and seed priming, which are potentially promising [76]. Although it has been reported that silymarin (Sm) or dopamine (DA) can help plants resist some stresses [18,19,32,77], the synergistic effects of Sm + DA on growth and productivity under Cd stress have not been studied to date. Increasing knowledge of the mode of action of protective antioxidants to cope with abiotic stresses is important to build credibility in agricultural practice. This research was performed to advance the understanding of mechanism(s), whereby Sm and DA, applied as seed soaking and foliar spraying, may exert a beneficial effect under cadmium stress conditions.

Considering the multiple functions of silymarin and dopamine in animals and humans, studies have emerged to show their potential effects, as potent antioxidants, on promoting growth and productivity in stressed plants, reinforcing their defensive systems. Although cadmium stress caused a decrease in the morphological, physiological, and biochemical parameters of beans, Sm or DA exogenous application (DA (SP) + Sm (FS)) or (Sm (SP) + DA (FS)) significantly alleviated this inhibition compared to untreated controls, which we report here for the first time (Figure 1, Figure 2, Figure 3, Figure 4, Figure 5, Figure 6, Figure 7 and Figure 8). Moreover, the highest positive effects were noted when silymarin and dopamine were used synergistically (silymarin seed priming + dopamine foliar spraying (Sm (SP) + DA (FS)). The precise role of silymarin as a secondary metabolite in enhancing the stressed plant performance has not been examined until now. Recent reports suggested that Sm, as a powerful antioxidant, can enhance plant growth and productivity through its accumulation in plants grown under stress to improve their self-defensive systems [18,19,78,79].

Growth and yield reduction in cadmium-stressed crops is common, and the present experiment confirmed a decrease in the growth and productivity characteristics (leaf number, leaf area, plant DW, pod number, and pod yield) of bean plants subjected to cadmium stress compared to unstressed, untreated plants. However, we noticed that Sm or DA exogenous application (DA (SP) + Sm (FS)) or (Sm (SP) + DA (FS)) significantly enhanced the growth and yield characteristics relative to the controls. Although significant growth and yield were achieved with Sm (SP) + DA (FS) under normal conditions, synergistic applications of (Sm (SP) + DA (FS) to cadmium-stressed bean plants enabled them to generate growth and yield traits similar to those in unstressed, untreated plants, as shown in Figure 1. The growth characteristics enhancement observed in silymarin-enriched bio-stimulant in maize [18] or DA-treated plants has been similarly reported earlier under different abiotic stresses, i.e., drought, nitrate, and alkalinity for some crops, such as cucumber, watermelon, and apple transplants [32,77,80]. The amelioration in the growth traits of cadmium-stressed bean plants in response to Sm or DA exogenous applications could be attributed to the accumulation of Sm and DA in stressed plants (Table 1), which might be involved in the mechanisms of non-enzymatic and enzymatic antioxidant responses (Figure 6, Figure 7 and Figure 8), which protect bean plants from damage [30,81]. Since it has a reducing power, dopamine can act as a potential antioxidant, which is effective in removing free radicals [82]. The previous literature also mentioned an interaction of dopamine with other phytohormones to regulate growth and enable plants to reinforce stress tolerance [33]. Dopamine maintained a higher level of auxin in plant tissues via the inhibition of IAA degradation [83]. Consequently, it can promote the stressed plant growth and development. Under salinity and drought stress, dopamine suppresses H_2_O_2_ production and enhances growth and development in *Malus* seedlings [32,36]. Additionally, dopamine has been recognized as a major regulator in the growth and development of *M. hupehensis* under nutrient deficiency, markedly alleviating this inhibition, as its level was significantly increased [84]. Under the adverse conditions of drought, DA enhances the growth of potato plants, and its level was also significantly increased [35]. Additionally, in this paper, exogenous Sm and DA synergistically maximized growth and yield and supported the conferment of positive impacts on cadmium-stressed common bean plants.

We noticed a simultaneous reduction in chloroplast pigments (chlorophylls and carotenoids) and efficiency of the photosynthetic machinery (photochemical activity and efficiencies of carboxylation (iCE) and PSII (*Fv/Fm*)) under cadmium stress. Photosynthetic efficiency is a precise indicator of the overall plant health, and exposure to environmental stresses reduces the photochemical quenching capacity within photosystem II [14,85]. Cadmium stress inhibits photosynthesis by reducing the activity of photosystem II, which causes an increase in cytotoxic compounds concentration [74]. Chlorophyll degradation and PSII photoinhibition observed in common bean plants could be attributed to the decrease in the plant water content (RWC) and MSI (Figure 3) required for photosynthesis and the negative influences of ROS on chloroplasts [86]. Previous reports proposed that the decline in chlorophyll content under stress is mainly attributed to H_2_O_2_ overproduction in plants [39]. Nevertheless, it was noticed that Sm or DA synergistically overturned this harmful effect and enhanced photosynthetic efficiency in common bean plants under cadmium stress (Figure 1 and Figure 4). The enhanced photosynthetic machinery in Sm or DA treated plants could be due to the abridged uptake and translocation of cadmium in common bean plant cells. In *Spinacia oleracea* isolated chloroplasts, dopamine controls the reduction in photosynthesis by functioning as an oxygen-reducing factor or a chemical analog of a suggested naturalistic mediator, which allows for transduction of energy during the photosynthesis process [33]. Additionally, directly or indirectly, DA can mitigate nutrient deficiency, drought, and salt, all of which restrict the performance of photosynthesis [32,36,84]. Our findings are in accordance with the findings of several investigators [17,36,80,82] who have also noticed the ability of dopamine to conserve the photosynthesis machinery within acceptable levels under different abiotic stresses, i.e., drought, nitrate, and alkalinity.

Synthesis of osmolytes, such as soluble sugars, proline, choline, GB, and K^+^ ion in plants, is an essential mechanism of protection against abiotic stress to prevent the proteins and cellular membranes from damage along with suppressing ROS [70,87,88]. In the current study, untreated cadmium-stressed plants showed high osmolyte accumulations (e.g., soluble sugars, proline, and GB; Figure 2) when compared to unstressed, untreated plants. However, Sm or DA (DA (SP) + Sm (FS)) or (Sm (SP) + DA (FS)) treated common bean plants under cadmium stress exhibited maximum accumulation of osmolytes as compared to untreated plants, contributing to an enhanced osmotic adjustment and providing a greater protection to the cells from cadmium toxicity. Soluble sugars, proline, and GB are the main organic osmolytes, which accumulate in most species of crop plants in response to stressors, including salinity, drought, and heavy metals [4,10,89,90]. Higher osmolyte synthesis appears to be the approach of common bean plants to tolerating cadmium stress (Figure 2) by osmotic adjustment, keeping the tissue turgor that allows plants to preserve growth and tolerate serious stresses [91,92]. Proline acts as an important osmolyte involved in enzyme protection, free radical scavenging, osmotic adjustment, and as a major source of energy and nitrogen [91,92,93]. As efficient compatible solutes, soluble sugars have been demonstrated to play pivotal roles in attenuating stress damage by conferring a desiccation resistance to plant cells and through osmotic adjustment [94]. The increase, in this study, of osmolytes (e.g., soluble sugars, proline, and GB) content under cadmium stress is substantiated with preceding outcomes [14,95,96]. The results of this research further explored the higher increment in osmolyte levels, stimulated by Sm or DA (DA (SP) + Sm (FS)) or (Sm (SP) + DA (FS)) treatments, which may protect the metabolism in common bean plants by suppressing excessive ROS induced by cadmium stress. The exposure of common bean plants to cadmium stress triggers a disturbance of the cellular redox balance and induces overproduction of oxidative stress biomarkers (i.e., O_2_^•−^, H_2_O_2_, and MDA; Figure 5), which may disrupt cellular metabolism, cause direct damage to membrane lipids, and interrupt the capacity for ion exchange of cell plasma membranes [97,98]. The findings of this research exhibited that exogenously used Sm or DA (DA (SP) + Sm (FS)) or (Sm (SP) + DA (FS)) decreased ROS levels, thus attenuating the cadmium stress effects. Our research outcomes confirmed those of Jiao et al. [16] who revealed decreased H_2_O_2_ content in watermelon exposed to chilling-induced stress. Comparable findings were also stated by Li et al. [36] in salt-stressed tea crabapple. Moreover, the enzymatic and non-enzymatic antioxidant activities are increased by oxidative stress to counteract its biomarkers (O_2_^•−^, H_2_O_2_, and MDA) and protect plant cells from damage of these biomarkers [99,100]. 

This study showed the cadmium-stress-stimulated defense (antioxidant) system of common bean plants by improving the activity of CAT, SOD, POD, and APX (Figure 7). Moreover, the levels of AsA and GSH (Figure 6) signified an involvement of these enzymes along with the AsA–GSH cycle in the antioxidative machinery in response to cadmium stress. Enzymatic antioxidants, particularly SOD, are the first line of defense against ROS. The anions of superoxide are resolved to O_2_ and H_2_O_2_ by superoxide dismutase, and then, the catalase and peroxidase decompose the intracellular hydrogen peroxide (H_2_O_2_) to molecular oxygen and H_2_O [16]. Exogenously utilized Sm or DA (DA (SP) + Sm (FS)) or (Sm (SP) + DA (FS)) restored these levels near to those explored in control-grown plants (Figure 7). This could be due to the potent antioxidative capacity of DA and Sm, both of which are water-soluble antioxidants. The possible cause of this phenomenon is the oxidation of DA to a free radical scavenger called melanin. This is close to previous data, whereby a silymarin-enriched bio-stimulant in maize [18] and DA exogenous application in apple and watermelon could raise the SOD, CAT, and POD activities and suppress increased H_2_O_2_ under chilling and salinity [16,80]. The endogenous levels of α-tocopherol, phenolic, flavonoids, GSH, and AsA were considerably increased under cadmium stress, and exogenously utilized Sm or DA (DA (SP) + Sm (FS)) or (Sm (SP) + DA (FS)) further raised these antioxidants, as seen in Figure 6. These potent antioxidants play pivotal roles in enhancing tolerance to abiotic stress impacts in plants through improving the scavenging ability by providing the electrons or hydrogen to cope with ROS [100,101,102]. This study showed that Sm or DA enhanced the accumulation of non-enzymatic antioxidants and reinforced the capacity of antioxidants in common bean plants under cadmium stress.

Cadmium-stressed common bean plants treated with Sm or DA (DA (SP) + Sm (FS)) or (Sm (SP) + DA (FS)) accumulated higher levels of polyamines (Put, Spd, and Spm). Moreover, the polyamine metabolic enzymes (ADC and ODC) were highly upregulated (Figure 8). Polyamines (PAs) are polycations with low molecular weights, which are ever present in all organisms [103]. They fundamentally mainly comprise spermidine (Spd), spermine (Spm), and putrescine (Put), the three most well-known species in plants [104]. They are elaborated in plant responses and their resistance to various stressors [92,105], and they maintain ion balance and redox homeostasis in plants [106]. The integrated systems of self-defense mechanisms of enzymatic and non-enzymatic antioxidants and osmolytes along with PAs, regulated by Sm or DA, not only assess the effectiveness in alleviating stress damage but also upregulate the physiological state to reach the integrity of defense responses in plants (Figure 5, Figure 6, Figure 7 and Figure 8). In this study, our results of a noticeable increment in endogenous PAs (Put, Spd, and Spm) and their metabolic enzyme (ADC and ODC) activities under cadmium stress are in line with the results in Refs [75,105] for wheat and in Ref [107] for *Vigna radiate*. Furthermore, the exogenous application of Sm or DA (DA (SP) + Sm (FS)) or (Sm (SP) + DA (FS)) further raised the levels and activities of ODC, ADC, Put, Spd, and Spm, while suppressing the activity of PAO. The findings of Gao et al. [108] signified that a decrease in PAs levels was detected in stressed maize plants, which may be due to increased PAO activity. This report [108] also indicated that the treatment of plants with PAs increased *SAMDC* activity and decreased PAO activity, thus enhancing the stress tolerance of plants. In addition, PAs treated plants exhibited upregulation of *ADC1*, *ADC2*, *ODC*, *SPDS*, and *SAMDC2* transcripts and a decrease in the relative expression of PAO genes (e.g., *PAO1*, *PAO2*, and *PAO3*), which resulted in decreased PAO activity and increased PAs levels in plant tissues [108]. Similarly, in this study, PAO activity was noticeably suppressed by Sm + DA treatment to increase PAs levels in *Phaseolus vulgaris* grown under Cd stress (Figure 8). Comparable outcomes were also reported [104] in watermelon exposed to chilling stress. Consequently, exogenous supplementation with Sm and DA is probably a potential strategy applied to Cd-stressed common bean plants to withstand the stress impacts by inducing the PAs enzymes metabolism activity to enhance PAs contents, thus reinforcing the plant defense system.

## 5. Conclusions

This research highlighted that exogenously supplemented Sm or DA could reinforce the resistance to cadmium damage in common bean plants by minimizing ROS accumulation, promoting enzymatic and non-enzymatic antioxidants and osmolyte accumulations to conserve cell membranes from stress damage. In addition to elevated endogenous polyamine (Put, Spd, and Spm) levels along with the polyamine metabolic enzyme (ADC and ODC) activities induced by silymarin and dopamine, they are probably the key factors capable of withstanding cadmium damage in common bean plants. Therefore, we propose that this synergistic action of dopamine and silymarin on cadmium stress damage resistance may provide new pathways for their implementation in crop production.

## Figures and Tables

**Table 1:** Impacts of seed and leafy treatments with silymarin (Sm) and dopamine (DA) on Sm and DA contents of cadmium-stressed *Phaseolus vulgaris* plants.

Treatment	Sm (mg kg^−1^ DW)	DA (μg kg^−1^ DW)
Control	11.1 ± 0.4 d	18.7 ± 0.02 d
DA (SP) + Sm (FS)	28.9 ± 0.9 b	18.8 ± 0.03 d
Sm (SP) + DA (FS)	15.7 ± 0.6 c	18.7 ± 0.03 d
Cadmium (Cd)	15.8 ± 0.6 c	29.4 ± 0.05 c
Cd + DA (SP) + Sm (FS)	36.2 ± 1.2 a	36.1 ± 0.07 b
Cd + Sm (SP) + DA (FS)	28.4 ± 0.8 b	45.2 ± 0.09 a

Same or different letters after mean ± SE within each column signify non-significant or significant differences, respectively, based on LSD test (*p* ≤ 0.05).

## Data Availability

The data presented in this study are available upon request from the corresponding author.

## Supplementary Materials

The following supporting information can be downloaded at: https://www.mdpi.com/article/10.3390/plants11223069/s1, Table S1: Preliminary study to identify the optimal concentrations of dopamine (DA) and silymarin (Sm) applied as seed soaking and foliar spray for Phaseolus vulgaris plants, as well as to identify Cd concentration that negatively affect plant growth without plant death.
