# Supplementary material for: Simultaneous Action of Silymarin and Dopamine Enhances Defense Mechanisms Related to Antioxidants, Polyamine Metabolic Enzymes, and Tolerance to Cadmium Stress in Phaseolus vulgaris
Source: Plants (Basel). 2022 Nov 12;11(22):3069. doi: 10.3390/plants11223069 (PMC9692805; doi:10.3390/plants11223069)
Supplement: Supplementary file 1 [file plants-11-03069-s001.zip › plants-1998740-supplementary.pdf]

Table S1. Preliminary study to identify the optimal concentrations of dopamine (DA) and silymarin (Sm) applied as seed soaking and foliar spray for *Phaseolus vulgaris* plants, as well as to identify Cd concentration that negatively affect plant growth without plant death.

Treatment	LNm plant^{-1}	LAr (cm^2 plant^{-1})	Plant DW (g)	PNm pot^{-1}	PYi (g pot^{-1})	TChls (mg g^{-1} FW)
Control	7.56±0.25a	872±71a	9.38±0.77a	26.8±0.72a	114±9a	2.79±0.08a
Cd-0.25	5.33±0.18b	594±43b	6.37±0.55b	14.5±0.39b	58±5b	1.42±0.05b
Cd-0.5	3.18±0.11c	270±14c	3.61±0.31c	5.33±0.18c	31±3c	0.82±0.03c
Cd-1.0	Plants died at flowering stage					
Control	7.56±0.25b	872±71b	9.38±0.77b	26.8±0.72b	114±9b	2.79±0.08b
DA$_{(1)}$SP	7.60±0.25b	876±72b	9.44±0.78b	26.9±0.73b	116±9b	2.82±0.08b
DA$_{(2)}$SP	8.52±0.31a	1088±84a	11.60±0.82a	31.9±0.80a	138±12a	3.08±0.12a
DA$_{(3)}$SP	8.49±0.30a	1082±83a	11.57±0.80a	31.8±0.78a	136±12a	3.06±0.11a
Sm$_{(1)}$SP	7.64±0.26b	879±74b	9.46±0.79b	27.0±0.74b	119±10b	2.88±0.09b
Sm$_{(2)}$SP	8.61±0.32a	1094±86a	11.68±0.85a	32.0±0.82a	142±14a	3.12±0.13a
Sm$_{(3)}$SP	8.58±0.31a	1089±85a	11.60±0.82a	31.8±0.80a	140±13a	3.10±0.12a
DA$_{(1)}$FS	7.64±0.26b	881±74b	9.48±0.80b	27.2±0.74b	120±10b	2.89±0.09b
DA$_{(2)}$FS	8.60±0.33a	1096±86a	11.66±0.84a	32.1±0.83a	141±13a	3.12±0.14a
DA$_{(3)}$FS	8.56±0.31a	1090±83a	11.61±0.82a	32.0±0.82a	139±12a	3.10±0.12a
Sm$_{(1)}$FS	7.74±0.28b	885±76b	9.49±0.81b	27.1±0.76b	121±10b	2.94±0.10b
Sm$_{(2)}$FS	8.68±0.34a	1098±87a	11.72±0.85a	32.4±0.84a	143±15a	3.14±0.14a
Sm$_{(3)}$FS	8.64±0.33a	1095±85a	11.70±0.82a	32.3±0.80a	140±14a	3.12±0.13a
Control	7.56±0.25d	872±71e	9.38±0.77d	26.8±0.72d	114±9e	2.79±0.08c
DA$_{(1)}$SP+Sm$_{(1)}$FS	7.98±0.27c	982±80d	10.4±0.79c	28.9±0.77c	128±11d	3.00±0.10b
DA$_{(2)}$SP+Sm$_{(2)}$FS	9.64±0.36a	1422±98b	14.8±0.92a	37.8±0.90a	170±17b	3.32±0.15a
DA$_{(3)}$SP+Sm$_{(3)}$FS	9.08±0.30b	1314±92c	14.0±0.88b	35.6±0.85b	158±14c	3.03±0.12b
Sm$_{(1)}$SP+DA$_{(1)}$FS	8.02±0.28c	988±81d	10.5±0.80c	29.0±0.79c	130±12d	3.00±0.11b
Sm$_{(2)}$SP+DA$_{(2)}$FS	9.84±0.39a	1496±101a	15.2±1.18a	39.2±1.2a	182±18a	3.38±0.17a
Sm$_{(3)}$SP+DA$_{(3)}$FS	9.05±0.30b	1310±91c	14.0±0.86b	35.3±0.83b	155±14c	3.00±0.11b

Same or different letters after mean ± SE within each column indicate non-significant or significant differences, respectively, based on LSD test ($p \leq 0.05$). LNm= leaves number, LAr= leaves area, DW= dry weight, PNm= pods number, PYi= pods yield, TChls= total chlorophylls, FW= fresh weight, Cd-0.25, Cd-0.5, and Cd-0.75= cadmium at 0.25, 0.5, and 0.75 mM, respectively, DA(1)FS, DA(2)FS, and DA(3)FS= dopamine applied as three foliar sprays at a concentration of 100, 200, and 300 µM, respectively, Sm(1)FS, Sm(2)FS, and Sm(3)FS= silymarin applied as three foliar sprays at a concentration of 200, 250, and 300 µM, respectively, DA(1)SP, DA(2)SP, and DA(3)SP= dopamine applied as seed soaking at a concentration of 100, 200, and 300 µM, respectively, Sm(1)SP, Sm(2)SP, and Sm(3)SP= silymarin applied as seed soaking at a concentration of 200, 250, and 300 µM, respectively.
